# Bisphenol-A in biological samples of breast cancer mastectomy and mammoplasty patients and correlation with levels measured in urine and tissue

**DOI:** 10.1038/s41598-021-97864-6

**Published:** 2021-09-16

**Authors:** Razieh Keshavarz-Maleki, Ahmad Kaviani, Ramesh Omranipour, Maryam Gholami, Mohammad Reza Khoshayand, Seyed Nasser Ostad, Omid Sabzevari

**Affiliations:** 1 Toxicology and Poisoning Research Centre, Tehran University of Medical Sciences, Tehran, Iran; 2 Department of Toxicology and Pharmacology, Faculty of Pharmacy, Tehran University of Medical Sciences, 1417614411 Tehran, Iran; 3 Department of Surgery, Imam Khomeini Hospital Center, Tehran University of Medical Sciences, Tehran, Iran; 4 Department of Surgical Oncology, Cancer Institute, Imam Khomeini Hospital Center, Tehran University of Medical Sciences, Tehran, Iran; 5 Breast Disease Research Center, Tehran University of Medical Sciences, Tehran, Iran; 6 Department of Drug and Food Control, Faculty of Pharmacy, Tehran University of Medical Sciences, Tehran, Iran

**Keywords:** Chemical biology, Toxicology

## Abstract

Endocrine disrupting chemicals (EDCs) are organic compounds that have estrogenic activity and can interfere with the endocrine system. Bisphenol-A (BPA) is one of these compounds which possess a potential risk for breast cancer. The aim of this research was to evaluate BPA concentration in both the urine and breast adipose tissue samples of breast cancer mastectomy and mammoplasty patients and study correlations of BPA levels in breast adipose tissue with urine samples in the both groups. Urine and breast adipose tissue samples from 41 breast cancer mastectomy and 11 mammoplasty patients were taken. BPA concentrations were detected using an ELISA assay. Urinary BPA concentrations were significantly higher in cancerous patients (2.12 ± 1.48 ng/ml; *P* < 0.01) compared to non-cancerous (0.91 ± 0.42 ng/ml). Likewise, tissue BPA concentrations in cancerous patients (4.20 ± 2.40 ng/g tissue; *P* < 0.01) were significantly higher than non- cancerous (1.80 ± 1.05 ng/g tissue). Urinary BPA concentrations were positively correlated with breast adipose tissue BPA in the case group (*P* < 0.001, R = 0.896). We showed that BPA was present in urine and breast adipose tissue samples of the studied populations. With regard to higher BPA mean concentration in cancerous patients than non-cancerous individuals in this study, BPA might increase the risk of breast cancer incidence.

## Introduction

Bisphenol-A (BPA) is an organic synthetic compound with a molecular weight of 228.29 g/mol belonging to the phenols’ group with the hydroxyl group directly attached to the aromatic ring^1^.This colorless solid compound is a monomer of polycarbonates and epoxy resins, which is used in a wide variety of products including dental products, protective coatings, thermal paper, baby bottles, medical equipment, electronics devices, compact discs, and food and beverages^2–4^. Increasing temperature and acidic or basic solutions can lead to degradation of polycarbonate plastics and epoxy resins, which results in BPA migration to ecosystems, food, and beverages^5^. Thus, diet is considered as the most important source of exposure to BPA^6^. Overall, the important routes of human exposure to BPA are ingestion, dermal contact, and inhalation since it has been found in the air, dust, water, and sewage leachates^7^.

Several studies monitored the presence of BPA in solid human tissues (adipose^8–10^, placenta^11^, liver and brain^12^), hair^13^ and body fluids (urine^14^, amniotic fluid^15^, blood^16^, sweat^17^, saliva^18^, cord blood^19^ and, breast milk^20^). The median levels of urinary concentration of BPA was reported 1.24 μg/l in American adults’ population and 1.25 μg/l in American childrens’ population by Lehmler et al.^21^. The reference dose (RFD) of BPA was announced 50 μg/kg/day by the United States Environmental Protection Agency (U.S.EPA)^22^. BPA is a xenoestrogen and functions as an endocrine disruptor with weak estrogenic activity that is named environmental. BPA has a structural similarity with 17-β-estradiol (E2) and directly activates estrogen-receptors (ERs)^23^. Due to its estrogenic-like properties, it is potentially related to many diseases such as breast and prostate cancers^6,24^. In Korean patients with breast cancer (70 cases, 82 controls), serum BPA concentration was not significantly higher than controls^25^. The BPA mean concentration was reported 2.39 ng/ml in the breast milk of French healthy women by Cariot et al.^20^.

BPA is a lipophilic compound and could accumulate in adipose tissue^10^. Breast adipose tissue is in close contact with breast epithelial cells; therefore, BPA can promote epithelial cell proliferation and induce neoplastic transformation in human breast cells^26^. It has been explored that BPA has proliferative potential in MCF-7 cells^27^.

Urinary BPA levels are typically used as a biomarker of BPA exposure and urine levels are dependent on recent exposure and might not be the suitable biomarker for the assessment of long-term exposure^28^. Adipose tissue might be an appropriate matrix for evaluation of chronic BPA exposure^9^ and a reliable biomarker of chronic exposure has not yet been recognized. Therefore, measurement of BPA in both samples can be helpful in affecting people's health and daily exposure can be compared to chronic exposure with measurement of BPA in both samples. However, only a few research has analyzed BPA levels in breast adipose tissue and urine samples in breast cancer patients. Hence, the present research was designed to study BPA concentration in both the urine and breast adipose tissue samples of breast cancer mastectomy and mammoplasty patients and to evaluate correlation of BPA levels in the breast adipose tissue with the urine samples in the both groups.

## Results

### Study population

All participants in this study were women. The mean age of cases was 53 years, ranging from 34 to 72 years. The body mass index (BMI) was estimated using height and weight parameters between 22.04 and 34.72 kg/m^2^, with a mean of 27 kg/m^2^. The majority of cases had a BMI of 25–30 kg/m^2^ (overweight) with the age group of 30 to 49 years. The mean age of controls was 47 years, ranging from 40 to 55 years. Control group members were younger than cases, but their BMI was similar to them (26.45 kg/m^2^). This study was performed on non-smokers who have never smoked in their lifetime because the urinary BPA concentration in smokers was higher than non-smokers in some studies^29,30^.

### Concentrations of BPA in human urine samples

Urine samples were spiked with three concentrations of BPA to evaluate the accuracy of the ELISA kit. The competitive ELISA demonstrated that on average 96.37% of the added BPA was recovered (Table 1).

**Table 1:** Recoveries of BPA in the urine samples (n = 3).

Sample	Added (pg/ml)	Found (pg/ml)	Recovery (%)
1	100	96.30	96.30
2	500	485	97
3	1000	958	95.80

Among the 41 analyzed samples, BPA was detected in 38 urine samples (92.68%). The level of total BPA (including free BPA and BPA conjugate) was 2.12 ± 1.48 ng/ml (mean ± SD) and a maximum concentration of 5.74 ng/ml. The highest BPA mean concentration was seen in the age group of 30 to 49 years (2.56 ± 1.50 ng/ml). BPA concentration was negatively correlated with age (*P* value < 0.05, r = − 0.373). In the BMI groups, the highest mean level of BPA was detected in the obese group (3.49 ± 1.69 ng/ml). There was no significant correlation between BPA concentration and BMI (*P* value > 0.05).

In control samples, BPA was found in 9 urine samples (81.82%) with a mean concentration of 0.91 ± 0.42 ng/ml. The maximum concentration of BPA was 1.82 ng/ml. As it is shown, the mean of the BPA concentration in the case group was significantly higher than the control group (*P* value < 0.01). The specifications of the study population are summarized in Table 2. Urinary BPA concentrations using medians and geometric means were summarized in Table 3.

**Table 2:** Characteristics of subjects and urinary BPA concentrations in cases and controls.

Characteristic	Number			Concentration of BPA in urine, Mean ± SD (ng/ml)		
	Cases N=41	Controls N=11	*P* value	Cases	Controls	*P* value
**Samples with detectable BPA; N (%)**	38 (92.68)	9 (81.82)		2.12 ±1.48	0.91 ±0.42	0.003**
**Age group (y); Mean± SD**						
**Among all samples**	52.68±9.17	48.18±4.62				
**Among samples with detectable BPA**	52.21± 9.26	48±5.12				
30-49; N (%)	17 (44.74)	5(55.56)		2.56±1.50	0.97±0.54	
50-59; N (%)	13 (34.21)	4 (44.44)		2.26±1.58	0.83±0.26	0.92
60-69; N (%)	5 (13.16)	–		1.11±0.42	–	
69-79; N (%)	3 (7.89)	–		0.71±0.24	–	
**BMI (Kg/m**^**2**^**); Mean± SD**						
**Among all samples**	26.55±2.82	25.91±3.27				
**Among samples with detectable BPA**	26.63± 2.90	26.43±3.41				
Recommended weight (18.5-25); N (%)	12 (31.58)	5 (55.56)		1.91±1.51	0.71±0.23	
Overweight (25-30); N (%)	22 (57.89)	3 (33.33)		1.98±1.36	0.93±0.26	0.97
Obese (30-35); N (%)	4 (10.53)	1 (11.11)		3.49±1.69	1.82±0	

** P < 0.01; comparison urinary BPA concentrations between cases and controls.

**Table 3:** BPA concentrations in cases and controls.

	Cases			Controls		
	Mean	Geometric Mean	Median	Mean	Geometric Mean	Median
Concentration of BPA in urine (ng/ml)	2.12	1.69	1.70	0.91	0.83	0.74
Concentration of BPA in breast adipose tissue (ng/g tissue)	4.20	3.50	4.11	1.80	1.50	1.65

### Concentrations of BPA in human tissue samples

BPA was detected in 30 out of the 41 (73.17%) breast adipose tissue samples, with a mean ± SD value of 4.20 ± 2.40 ng/g tissue (0.84 ± 0.48 ng/ml). The highest concentration of BPA was 9.90 ng/gtissue. No significant correlation was found between BPA level and age (*P* value > 0.05). BPA concentration was slightly higher in the obese group compared to non-obese (*P* value > 0.05). No significant correlation was observed between the BPA level and BMI (P-value > 0.05).

BPA was detected in 6 breast adipose tissue samples (54.54%) in the control group with the mean concentration of 1.80 ± 1.05 ng/g tissue (0.36 ± 0.21 ng/ml). The highest concentration of BPA was 3.11 ng/g tissue. BPA levels in controls were significantly lower than cases (*P* value < 0.01). Table 4 shows the specification of the study population. Adipose tissue BPA concentrations using medians and geometric means were summarized in Table 3.

**Table 4:** Characteristics of subjects and tissue BPA concentrations in cases and controls.

Characteristic	Number			Concentration of BPA in breast adipose tissue, Mean ± SD (ng/g tissue)		
	Cases N = 41	Controls N = 11	*P* value	Cases	Controls	*P* value
**Samples with detectable BPA; N (%)**	30 (73.17)	6 (54.54)		4.20 ± 2.40	1.80 ± 1.05	0.008**
**Age group (y); Mean ± SD**						
**Among all samples**	52.68 ± 9.1	48.18 ± 4.62				
**Among samples with detectable BPA**	51.37 ± 9	47.17 ± 6.11				
30–49; N (%)	14 (46.67)	4 (66.67)		4.95 ± 2.30	1.50 ± 1.05	
50–59; N (%)	10 (33.33)	2 (33.33)		4.20 ± 2.60	2.40 ± 0.95	0.98
60–69; N (%)	4 (13.33)	–		2.85 ± 1.40	–	
69–79; N (%)	2 (6.67)	–		1.60 ± 1.20	–	
**BMI (Kg/m**^**2**^**); Mean ± SD**						
**Among all samples**	26.55 ± 2.82	25.91 ± 3.27				
**Among samples with detectable BPA**	26.93 ± 3.05	26.91 ± 4.16				
Recommended weight (18.5–25); N (%)	9 (30)	3 (50)		4.45 ± 2.65	1.55 ± 1.40	
Overweight (25–30); N (%)	17 (56.67)	2 (33.33)		3.75 ± 2.30	2.30 ± 0.85	0.48
Obese (30–35); N (%)	4 (13.33)	1 (16.66)		5.60 ± 2.25	1.55 ± 0	

** P < 0.01; comparison tissue BPA concentrations between cases and controls.

### Correlation of BPA concentrations in case and control groups

**Figure 1:**
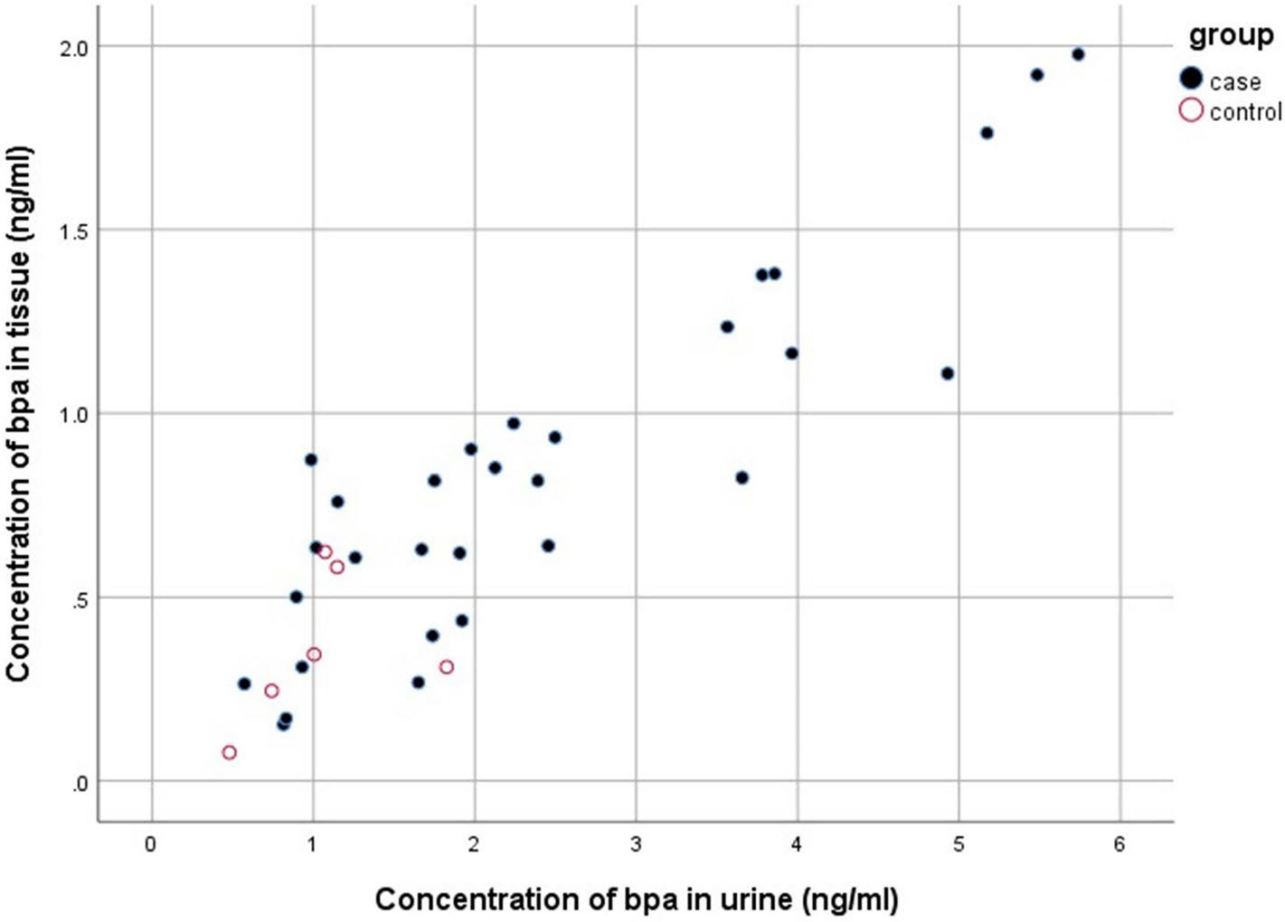
Correlation of BPA concentrations in urine with adipose tissue in case and control groups.

BPA concentrations in urine were positively correlated with BPA concentrations of adipose tissue (*P* value < 0.001, R = 0.896) in the case group (Fig. 1).

No significant correlation was observed between the urinary BPA concentrations and tissue BPA concentrations (*P* value > 0.05) in the control group (Fig. 1).

### The effects of different variables on the breast cancer

The effects of different variables on the breast cancer were examined using Bayesian logistic regression. In Model I, all variables are included in a unique model. The result of this Model is not statistically valid due to the strong collinearity of BPA concentrations in urine and tissue (r = 0.898, *P* value < 0.001), which leads to the non-significance effect of the two variables. Model II refers to the univariate analysis of different variables on the cases in which the effects of both BPA concentrations in urine and tissue were statistically significant when the effects of age and BMI were not adjusted. Finally, in Model III and IV, the effects of BPA concentration in tissue and urine were separately evaluated when the effects of age and BMI were adjusted.

The result of Model II, III, and IV shows that increasing the BPA concentrations in urine and tissue significantly increased the odds of breast cancer (Table 5).

**Table 5:** The effects of the different variables on the breast cancer.

	Model I		Model II		Model III		Model IV	
	OR (95% CI)	*P* value	OR (95% CI)	*P* value	OR (95% CI)	*P* value	OR (95% CI)	*P* value
Age	1.11(0.99,1.24)	0.088	1.07(0.98,1.16)	0.152	1.1(0.98,1.23)	0.118	1.13(1.01,1.26)	0.028
BMI	0.91(0.66,1.26)	0.557	1.05(0.83,1.32)	0.679	0.98(0.74,1.3)	0.900	0.84(0.6,1.16)	0.272
Concentration of bpa in tissue (ng/ml)	7.21(0.2,259.03)	0.273	21.23(1.21,369.8)	0.037	54.96(2.08,1372.55)	0.018	–	–
Concentration of bpa in urine (ng/ml)	3.1(0.7,14.18)	0.143	3.58(1.15,11.71)	0.035	–	–	10.59(1.62,65.7)	0.010

OR: odds ratio; CI: Credible interval.

## Discussion

Biomonitoring projects confirmed widespread human exposure to BPA in the world and have revealed the association of BPA concentration with various adverse health effects. To our knowledge, this is the first study to evaluate BPA levels of both urine and breast adipose tissue samples in breast cancer mastectomy and mammoplasty patients, using the ELISA method in Iranian populations.

BPA has a short half-life and is metabolized to its conjugates quickly and excreted through urine^31^. In our study, BPA concentration mean was measured 2.12 ng/ml in patients' urine samples, which was significantly different from the control group (*P* value < 0.01). The geometric mean urinary concentration of BPA has been detected 1.06 ng/g creatinine in breast cancer patients and 1.16 ng/g creatinine in non-breast cancer individuals by Morgan et al. that they observed significant difference between groups^32^. In addition, Parada et al. reported that urinary BPA concentration median was 1.20 ng/ml in breast cancer and 1.30 ng/ml in non-breast cancer subjects that there was no significant difference between groups^33^. In a study by Trabert et al. in Poland, however, urinary BPA-glucuronide (BPA-G) concentration and its association with postmenopausal breast cancer risk was evaluated using HPLC/MS/MS method. The geometric mean of BPA-G levels was higher in breast cancer cases (4.11 ng/mg creatinine) than controls (3.92 ng/mg creatinine). Although, BPA concentration was higher in breast cancer patients, but they observed no relation between postmenopausal breast cancer and urinary BPA-G levels at the time of diagnosis^34^. We found that in the urine sample of the mammoplasty control group, the mean concentration of BPA was 0.91 ng/ml with a detection frequency of 81.82%. Pirard et al. reported that urinary BPA concentration mean was 2.40 ng/ml (96.9% detection rate) in Belgium healthy women^35^, which was higher than our findings. In Ahmadkhaniha et al. assay in Iran, urinary BPA concentration and its association with type-2 diabetes mellitus was evaluated using GC/MS. BPA was determined with a mean concentration of 2.9 ng/ml (49.8% detection rate) in type-2 diabetes mellitus patients and 0.5 ng/ml (50.2% detection rate) in healthy individuals as a control group, which was lower than our control group (mean concentration of 0.91 ng/ml). They observed a significant positive correlation between BPA level and prevalence of type-2 diabetes mellitus^36^.

Since the evaluation of BPA concentrations in tissues needs surgery or biopsy procedures, the study on human tissue sample is often difficult and hardly acceptable. However, it seems very much helpful and informative, due to its association with various diseases and adverse health outcomes. Because of lipophilic property, BPA is detectable in fatty tissues or fluids^10^.

One of the limitations of our study was the low access to control samples due to the limited number healthy people referring to this center for the procedure.

We found that BPA concentration mean in breast adipose tissue was 4.20 ng/g tissue with 73.17% detection frequency in cases. Statistical difference was found between the mean concentrations of BPA in the cases and controls (*P* value < 0.01). There were only two studies that have measured BPA in breast adipose tissue^26,37^ and a few studies which have evaluated BPA in peripheral adipose tissue^8–10,12^. Reeves et al. have detected low rates of BPA levels in breast adipose tissue of breast cancer patients (26.1%; mean concentration of 0.71 ng/g tissue) using HPLC–ESI–MS/MS method^26^. It shows that BPA concentrations in their tissue samples were much lower than our study that these differences in concentrations may be attributable to variations among individuals in BPA exposure, which is significantly influenced by age, BMI, lifestyle factors, race/ethnicity, eating habits, and other sociodemographic characteristics. Venisse et al. measured BPA levels in breast adipose tissue of cancerous patients with a mean equal to 5.66 ng/g tissue using LC–MS/MS method^37^. In two peripheral adipose tissue studies from America and Belgium, BPA was determined with a mean concentration of 3.95 ng/g tissue (90% detection rate) from liposuction patients using HPLC–MS/MS method^8^ and 3.78 ng/g tissue (100% detection rate) from autopsy study using GC-ECNI/MS method^12^, respectively. BPA concentration mean was 1.80 ng/g tissue (54.54% detection rate) from mammoplasty patients’ tissue samples in our study, which is lower than their reported levels.

Urinary BPA levels were about 2.5-fold higher than adipose tissue concentrations in both mastectomy and mammoplasty groups. In addition, BPA concentrations in urine were positively correlated with BPA concentrations of adipose tissue (*P* < 0.001, R = 0.896) in the case group. Artacho-Cordon et al. measured BPA levels in peripheral adipose tissue (mean concentration of 0.6 ng/g tissue) and urine (mean concentration of 1.14 ng/ml) samples from trauma patients. They observed no correlation between BPA levels in urine and adipose tissue samples^9^.

In our assay, BPA concentrations were inversely associated with age and BPA levels decreased in the upper age groups, which was in line with the study of Lang et al.^4^. The highest BPA concentrations were seen in the obese group in urine and tissue samples of both cases and controls. Since the diet is the most important route of exposure to BPA, increased concentration of BPA in the obese group can be attributed to more food consumption and subsequently higher intake of BPA, compared to normal-weight ones. It should be noted that obesity is evaluated as a risk factor for breast adipose tissue inflammation that has been offered an indirect outcome of obesogenic contaminants^38^. Similarly, in Reeves et al. study, the highest breast adipose tissue concentrations of BPA were observed in the obese group^26^.

Comparability of monitoring data is necessary to environmental exposure assessment involving the management of environmental risks. Based on the previous studies and the present study, analysis of urinary BPA is a valuable tool to evaluate BPA exposure for the following reasons: 1) Collection of the urine sample is easy and non-invasive, 2) Rapid biotransformation of BPA and excretion into the urine, 3) Short half-life of BPA (< 6 h), 4) Reflection of all routes and recent BPA exposure into urine. The log of the octanol–water partition coefficient (Kow) of BPA is 3.64;^8^; hence, BPA is a potential risk for bioaccumulation and can be distributed to adipose tissue. Due to the short half-life of BPA, however, urine may not reflect prolonged exposure to BPA, and adipose tissue levels may be a more suitable source for assessment of mid- to long-term exposure. We showed that BPA was present in the urine and breast adipose tissue samples of the studied Iranian population and exposure to BPA might increase the risk of breast cancer incidence. However this study experienced some limitations and the presented data should be interpreted with care; since the sample size is fairly small and a single urine measurement was employed. Nevertheless, due to chronic exposures to BPA and prior longitudinal assessments, employment of a single-spot sample could be considered reasonable^39^. If the potential role of environmental exposures to BPA in the development of breast cancer would be confirmed by future studies, reducing BPA exposure may play an important role in the prevention and reduction of breast cancer incidence. On the other hand, adipose tissue is important in tumor microenvironments and is currently identified as a main agent in the development, growth, and promotion of cancer^40^. Breast adipose tissue inflammation intervenes in early-stage breast cancer^41^. An interesting study has conducted by Giulivo et al. revealed that low doses of BPA might induce secretion of inflammatory mediators such as interleukin-6 (IL-6) and tumor necrosis factorα(TNF-α)^5^. With regard to the importance of exposure-disease relationships, further research is needed to investigate the correlation between BPA exposure with growth, and progression of breast cancer.

## Conclusion

In this dissertation, we measured BPA concentrations in urine and breast adipose tissue samples obtained from breast cancer patients and mammoplasty procedures. These findings are important for public health in order to know the BPA levels in the population. Data presented here illustrate that urinary BPA concentrations were positively correlated with BPA concentrations in breast adipose tissue which evidence that BPA can accumulate in breast adipose tissue due to its lipophilic and bioaccumulation properties. Even with the limitation of obtaining more control subjects in this study, BPA mean concentrations in cancerous patients were significantly higher than non-cancerous individuals, which could indicate the presence of BPA in breast adipose tissue might increase the risk of breast cancer incidence. Notably, it is recommended to reduce the consumption of canned foods and replace plastic containers with glass-wares to heat foods. This information is of interest, because BPA is a ubiquitous environmental pollutant and an endocrine-disrupting compound which may interfere in the pathogenesis of hormonal-dependent cancers, and breast cancer in particular.

## Materials and methods

### Chemicals and reagents

Environmental Estrogen ELISA kit “Bisphenol A” was purchased from Detroit R&D (Inc, Detroit, MI). Bisphenol A (≥ 99% purity) obtained from Sigma-Aldrich (St. Louis, MO, USA).

### Study design and sample collection

Forty-one human urine and breast adipose tissue samples were obtained from breast cancer mastectomy patients and eleven control samples were taken from reduction mammoplasty patients during surgery at the university hospital of Imam Khomeini, Tehran, Iran by simple randomized sampling from July 2018 to December 2019. Questionnaire forms, including distribution of demographic and health characteristics, lifestyle factors, current residence and eating habits, were filled through an in-person interview. All subjects confirmed the questionnaire content with signature. The Ethical approval was obtained from Ethics Committee of Tehran University of Medical Sciences. Exclusion criteria were a family history of breast cancer, smoking and underlying diseases (e.g., cardiovascular diseases, diabetes mellitus, respiratory disease, liver disease, and thyroid disease) in both cases and controls. In breast cancer cases, an adipose tissue sample was collected from the proximity of the tumor. A standard materials list was checked to potential sources of BPA contamination and was removed before sample collection to minimize the possibility of contamination from surgical instruments^26^. Surgeons took care not to touch samples with any plastic equipment. They used a clean scalpel to remove tissue. First Morning spot urine and adipose breast tissue samples were collected in BPA-free glasses, coded, and were stored at − 20 °C until analysis. Urine samples were taken in cases and controls groups one day before surgery.

### Environmental estrogen ELISA kit

The enzyme-linked immunosorbent assay (ELISA) is a commonly used analytical biochemistry assay and this method is very sensitive, quantitative and accurate^42,43^. The competitive ELISA kit (Detroit R&D) was used for the determination of BPA levels in urine and tissue samples. The limit of detection (LOD) was less than 10 pg/ml (0.065 pg/g tissue). This ELISA kit works based on competition between the BPA epitope and the BPA-HRP (BPA-horseradish peroxidase) conjugate for a limited number of anti-BPA antibody binding sites coated on the bottom of the ELISA plate wells. Thus, the amount of the BPA conjugate, which can bind to each of the wells, is reversely proportional to the concentration of BPA in the standard or sample. When TMB (Tetramethylbenzidine) is added, it reacts with the HRP in the well and is produced a blue color that indicates the amount of the conjugate bound to each well. Following the addition of sulfuric acid, a yellow-colored product is obtained. Then, the optical absorbance is read at 450 nm by a plate reader. This ELISA kit is specific for measurement of BPA levels. The specificity test of the BPA ELISA was investigated using authentic BPA and a panel of bisphenols and related chemicals. The cross-reactivity tests with BPF, BPS and resveratrol demonstrated minimal cross-reactivity: BPA 100%, BPF < 0.01%, BPS < 0.01%, Resveratrol < 0.01%.

### Preparations of urine samples

Urine samples were diluted fourfold with 1X sample dilution buffer and were centrifuged to remove any precipitates. Then, 100 μL of the samples were added to each well of the 96-well plate and BPA levels were measured according to the manufacturer's instruction. Each sample was assayed in triplicate and the absorbance readings from the samples were averaged. Extraction recoveries were calculated for three urine samples by spiking a known amount of BPA into the urinary samples, and the recoveries ranged were displayed in the result section.

### Preparations of adipose breast tissue samples

250 mg of tissue was homogenized with 1 mL of H2O with a mechanical homogenizer, and the homogenate was acidified by adding 2 μL of acetic acid. Following extraction with an equal amount of ethyl acetate, it was vortexed, and the organic phase was collected. This extraction procedure was repeated 2–3 times, and the organic phases were merged. The organic phase was dried under nitrogen gas, and the pellet was dissolved using 5 μL of ethanol. Consequently, 125 μL of 1× sample dilution buffer was added to the resuspended extract and was centrifuged at 10,000 rpm for five minutes at room temperature. The supernatant was used to measure BPA concentration by the ELISA method according to the manufacturer's instruction. Each sample was assayed in triplicate and the absorbance readings from the samples were averaged.

### Performing the assay of samples

According to the instructions of the manufacturer, 100 μl of the samples and 100 μl of diluted BPA-HRP conjugate were added to each well of plate and the plate was incubated at room temperature for two hours. Then, the plate was washed three times with 400 μl of diluted wash buffer per well. The plate was dried onto some paper toweling at the last of the three wash cycles. To all the wells, 200 μl of TMB substrate was added and the plate was incubated at room temperature for 15–30 min. Finally, 50 μl of 2 N sulfuric acid was added and the optical absorbance was read at 450 nm by a plate reader. A standard curve was prepared for the analysis and BPA concentrations were determined utilizing the standard curve. The intra- and inter-assay coefficients of variance were 7.3% and 8.7%, respectively.

### Statistical analysis

Data were presented as means ± standard deviation (SD). Correlations were assessed with Spearman correlation coefficients. Wilcoxon rank sum test was used for comparison of the mean BPA levels between cases and controls. Generalized Linear Model was used to compare mean urinary and tissue concentrations among the age groups and the BMI groups between cases and controls. According to the small sample size, the Bayesian logistic regression was applied to handle the data separation (quasi or completed separation). A value of *P* < 0.05 was considered to be significant. Data were analyzed using SPSS software for Windows (Version 16.0. Chicago, SPSS Inc.) and R software version 4.0.2. [ 44].

### Statement of ethical approval

This research was approved by the research committee of Tehran University of Medical Science and institutional ethics committee with approval code of No.IR.TUMS.PSRC.REC.1395.1879. This research committee confirms that all research was performed in accordance with relevant guidelines/regulations and informed consent was obtained from all participants and/or their legal guardians.

### Consent to participate

Informed consent was obtained from all individual participants included in the study.

### Consent to publication

Informed consent was obtained from all individual participants for whom identifying information is included in this article.

## Data Availability

All data generated or analyzed during this study are included in this submitted article.
